# Meta-analysis of dietary chicory (*Cichorium intybus* L.) modulation of the growth performance of broiler chickens

**DOI:** 10.1016/j.psj.2026.107956

**Published:** 2026-10-15

**Authors:** Christiaan Jacobus Smit

**Affiliations:** Department of Agriculture and Animal Health, College of Agriculture and Environmental Sciences, University of South Africa, Florida Science Campus, Florida Park, South Africa

**Keywords:** Body weight gain, Chicory, Feed conversion ratio, Feed intake, Phytotherapy

## Abstract

Antibiotic growth promoters are increasingly restricted in broiler production over concerns about antimicrobial resistance, prompting interest in phytotherapies such as chicory. However, individual trials on dietary chicory in broilers report inconsistent effects on growth performance, limiting confident commercial translation. This study synthesized data from 2010 to 2025 to estimate the pooled effect of chicory on feed intake (FI), body weight gain (BWG), and feed conversion ratio (FCR) in broiler chickens. A random-effects model computed standardized mean differences (SMD) with 95% confidence intervals, and subgroup, meta-regression, and publication bias analyses were conducted for outcomes with significant pooled effects and heterogeneity (*I*^2^ > 50%). Dietary chicory increased FI (SMD = 0.420; 95% CI: 0.104 to 0.736; *p* = 0.009; *I*² = 94.29%) and BWG (SMD = 1.000; 95% CI: 0.718 to 1.282; *p* < 0.001; *I*² = 93.24%) and lowered FCR (SMD = -0.450; 95% CI: -0.660 to -0.240; *p* < 0.001; *I*² = 86.07%), relative to control diets. The inclusion level and growth stage moderated FI. Continent, inclusion level, form, and sex moderated BWG. Continent, inclusion level, form, route, and sex moderated FCR. Fail-safe numbers for FI (749), BWG (5,535), and FCR (770) all exceeded their robustness thresholds. Dietary chicory improves growth performance, supporting its potential as an alternative to antibiotic growth promoters. However, heterogeneity left unexplained by the tested moderators highlights the need for standardized dosing and reporting in future trials to enable commercial adoption.

## Introduction

Through enhanced genetics and nutrition, the broiler chicken industry has witnessed rapid growth, which supports food security by supplying the nutrient requirements of a growing human population (Korver, 2023; Tavárez and Solis de los Santos, 2016). Antibiotics have traditionally been utilized in broiler chicken production at subtherapeutic dosages to improve nutrient utilization and feed efficiency. Nonetheless, the correlation between antimicrobial resistance and indiscriminate antibiotic usage (Bungau et al., 2021) has prompted restrictions by relevant authorities in many countries (Beber et al., 2025). This has led to research into sustainable alternatives such as synbiotics (Khosravi et al., 2025), organic acids (Chukwudi et al., 2025), bacteriophages (Wanasawaeng et al., 2025), phytotherapies (Rafeeq et al., 2022), probiotics, and prebiotics (Rehman et al., 2020) as replacements for antibiotic growth promoters to maintain or enhance broiler chicken productivity and health.

Phytotherapies, through their bioactive compounds, provide antimicrobial and immunomodulatory effects without the viability constraints of probiotics (Ramlucken et al., 2021), the palatability issues of organic acids (Chukwudi et al., 2025), the heat sensitivity of enzymes (Boltz et al., 2020), the host specificity of bacteriophages (Cao et al., 2022), or the proteolytic instability of antimicrobial peptides (Bhandari et al., 2026). One such phytotherapy is chicory (*Cichorium intybus* L.), a perennial herb that contains inulin, minerals, vitamins, tannins, flavonoids, fatty acids, phenolic compounds, and reducing sugars (Akram et al., 2025). Chicory inulin has been linked to boosting *Lactobacillus* spp. populations and immune system proteins in broiler chickens (Wu et al., 2019). These characteristics position chicory as a functional ingredient with potential to improve gut and immune health and, by extension, growth performance in broiler chickens.

Nevertheless, individual trials evaluating dietary chicory in broilers have produced inconsistent results. For instance, Abbasi et al. (2024) and Tufarelli et al. (2023) reported increased feed intake (FI) and body weight gain (BWG) with a reduced feed conversion ratio (FCR) in male and mixed-sex Ross 308 birds fed diets containing 21.3 g/kg and 1 g/kg chicory root powder, respectively, after 42 days. In mixed-sex Cobb 500, dietary inclusion of 3 g/kg chicory extract increased BWG while decreasing FI and FCR (Abo Ghanima et al., 2023). Nonetheless, 10 g/kg chicory root powder did not vary the FI, BWG, and FCR of Ross 308 after 24 days (Izadi et al., 2013). This heterogeneity, driven by individual experimental variation, makes it difficult to define a generalizable inclusion strategy from any single study. Such inconsistency carries direct commercial consequences. For instance, without a reproducible dose-response relationship, feed manufacturers could be unable to formulate chicory into commercial diets, exposing producers to the risk of inconsistent flock performance, wasted feed costs, or even inadvertent growth depression if inclusion levels are poorly calibrated. This uncertainty is compounded by the small sample sizes and limited statistical power typical of individual trials (Pesti et al., 2023), which could constrain the precision of effect estimates and limit their extrapolation to commercial production systems.

Thus, a quantitative synthesis is needed to reconcile conflicting findings and establish the true magnitude and consistency of chicory's effects on broiler growth performance. This meta-analysis, therefore, aggregates data from published trials to estimate the overall effect of dietary chicory supplementation on growth performance outcomes and to identify moderators driving the observed variability. This study hypothesized that dietary chicory would enhance growth performance and that the magnitude of these responses would be predicted by continent, inclusion level, growth stage, form, inclusion route, sex, and strain. By consolidating the existing evidence base, this work aims to provide poultry nutritionists and feed formulators with a reliable basis for incorporating chicory into broiler diets as alternatives to antibiotic growth promoters at a commercial scale.

## Material and methods

### Criteria for inclusion and exclusion with search strategy

A systematic literature search from 2010 to 2025 was performed following the protocol of PRISMA. The search was performed on the databases of AGORA, Google Scholar, PubMed, Scopus, and Web of Science. The search string applied across all databases was chicory or “*Chicorium intybus*” AND “broiler chicken” AND “growth performance” OR “feed intake” OR “body weight gain” OR “feed conversion ratio”. Deduplication of all retrieved records was done via Covidence’s systematic review web-based software platform before screening. Selection of studies was based on the PICO framework with broiler chickens in unchallenged trials serving as the population. However, in studies that reported separate means for non-challenged and challenged trials, we retained the means for non-challenged controls and treatments. The intervention involved administering chicory through either feed or drinking water in any form. The comparison included a control group that was fed the basal diet without any additives, as well as a group fed chicory. The outcomes measured were growth performance [feed intake (FI; g), body weight gain (BWG; g), or FCR (g/g)]. Fig. 1 summarizes the selection and exclusion procedure. Two reviewers assessed the included studies independently with the SYRCLE risk-of-bias tool for animal studies (Hooijmans et al., 2014), covering ten domains across selection, performance, detection, attrition, reporting and other biases. Each domain was rated low, unclear or high risk, and disagreements resolved by consensus.

**Fig. 1:**
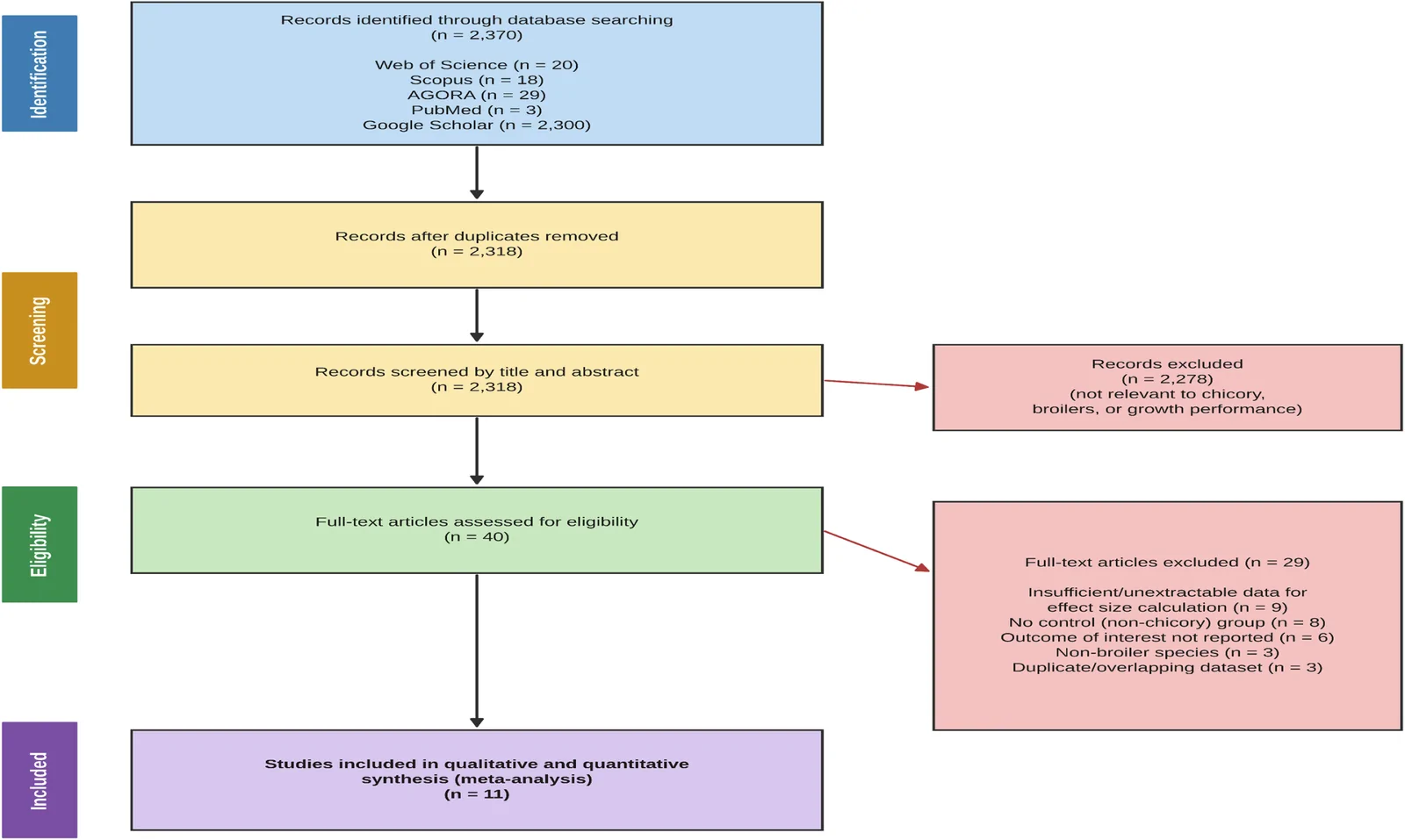
PRISMA flow diagram describes the literature search strategy and study selection criteria.

### Data extraction

Table 1 presents information on the surname of the first author, year of publication, continents, number of birds, number of treatments, strain, sex, inclusion level, route, form, and outcomes extracted from the 11 studies that met the inclusion criteria. The Excel sheet used for analysis after conversion into CSV files contains data on the growth stage, control group mean, control standard deviation, control sample size, experimental group mean, experimental standard deviation, and experimental sample size. Where standard error (SE) was reported in a trial study in place of standard deviation (SD), the SE values were converted into SD using SD=SE×√n where *n* is the number of replicates in each treatment group.

**Table 1:** Description of studies that met the inclusion criteria.

No	Authors	Continent	NB	NT	Strain	Sex	Inclusion level (g/kg)	Inclusion route	Form	Outcomes
1	Abbasi et al. (2024)	Asia	80	2	Ross 308	Male	0-21.3	Feed	Powder	1, 2, 3
2	Selionova et al. (2024)	Europe	72	2	Smena-9	Mixed	0-0.56	Feed	Extract	2
3	Yousaf et al. (2024)	Asia	60	2	Hubbard	Mixed	0-20	Water	Powder	1, 2, 3
4	Tufarelli et al. (2023)	Europe	160	2	Ross 308	Mixed	0-10	Feed	Powder	1, 2, 3
5	Gazwi et al. (2022)	Africa	120	2	Ross 308	Mixed	0-0.5	Feed	Extract	1, 2, 3
6	Khoobani et al. (2019)	Asia	180	4	Ross 308	Mixed	0-2	Feed	Powder	1, 2, 3
7	Mousa et al. (2017)	Africa	100	2	Ross 308	Mixed	0-15	Feed	Powder	1, 2, 3
8	Saeed et al. (2015)	Asia	120	4	Unknown	Mixed	0-15	Water	Extract	1, 2, 3
9	Taraz et al. (2015)	Asia	80	4	Ross 308	Male	0-0.35	Feed	Extract	1, 2, 3
10	Izadi et al. (2013)	Asia	120	3	Ross 308	Male	0-30	Feed	Powder	1, 2, 3
11	Liu et al. (2011)	Europe	256	4	Ross 308	Male	0-60	Feed	Extract	1, 2, 3

NB = number of birds; NT = number of treatments; 1 = feed intake (g); 2 = body weight gain (g); 3 = feed conversion ratio g/g);.

Where studies reported inclusion levels as a percentage or parts per million (ppm), values were converted to g/kg by multiplying by 10 or 0.001, respectively. Where inclusion levels were noted in mg/kg, values were converted to g/kg by dividing by 1000. When studies included multiple levels of chicory against a control group, each treatment level was noted as a separate effect size, with the shared control group divided proportionally across levels to avoid the unit-of-analysis error associated with the control being counted twice following Cochrane’s recommendation (Garritty et al., 2024).

### Data analysis

The OpenMEE software, which is built in R, was used for data analysis with the random-effects model used to estimate the pooled effects as a standardized mean difference (SMD) with a 95% confidence interval (CI). For the FI, BWG, and FCR, the SMD was computed as the difference between the chicory treatment and the control. Thus, a negative or positive SMD signifies a decrease or an increase moderated by chicory relative to control. A random-effects model with restricted maximum likelihood (REML) estimation was used for all analyses with the following statistical model:

$$Y_{ij}=\mu +T_{i}+u_{i}+e_{ij}$$

where *Y*_ij_ is the observed effect size, μ is the overall mean, *T*i is the fixed effect of chicory treatment relative to the control, *u*_i_ is the random effect, and *e*_ij_ is the residual sampling error.

The Cochran *Q* test and its associated *p*-value and heterogeneity (*I²*) were used to describe between-study variation. Subgroup, meta-regression, and publication bias analyses were evaluated for growth performance indices with significant pooled effects (*p* < 0.05) and *I²* (> 50%). Subgroup and meta-regression analysis were evaluated based on the following moderators: continent, inclusion level (g/kg), growth stage (starter, grower, finisher, or overall), form (powder or extract), route (diet or drinking water), sex (male or mixed), and strain (Hubbard, Ross, Smena-9, or unknown). The meta-regression used the R² analog to note the extent to which moderators have explained the variance. To evaluate the robustness of the results, publication bias was measured using funnel plots and Rosenberg’s weighted fail-safe N (*N*_fs_). Results were adjudged robust against publication bias if the calculated *N_fs_* exceeded the standard threshold of *5N_study_* + 10 (where *N_study_* is the number of included studies). The leave-one-out analysis was used to evaluate the sensitivity of the pooled estimates with significance for all tests set at *p* < 0.05.

## Results

### Methodological quality assessment

No study was rated low risk across all domains. Selective outcome reporting was low risk in all 11 studies (100%), and baseline similarity (45%) and other bias (55%) were low in about half. Reporting was most deficient for sequence generation (91% unclear), allocation concealment (91% unclear) and blinding of caregivers (100% unclear) and assessors (91% unclear). Random housing (18% low) and complete outcome data (18% low) were rarely documented (Supplementary Figures 1 and 2).

### Effect of chicory on growth performance of broiler chickens

The pooled estimate indicated that dietary chicory increased (SMD = 0.420; 95% CI 0.104 to 0.736; *p* = 0.009; *I²* = 94.29) FI relative to the control group (Fig. 2). Similarly, dietary chicory increased BWG (SMD = 1.000; 95% CI 0.718 to 1.282; *p* < 0.001; *I²* = 93.24) compared to the control group (Fig. 3). Regarding FCR, broiler chickens fed diets containing chicory had a lower (SMD = −0.450; 95% CI −0.660 to −0.240; *p* < 0.001; *I*² = 86.07%) FCR compared to those fed diets without chicory (Fig. 4).

**Fig. 2:**
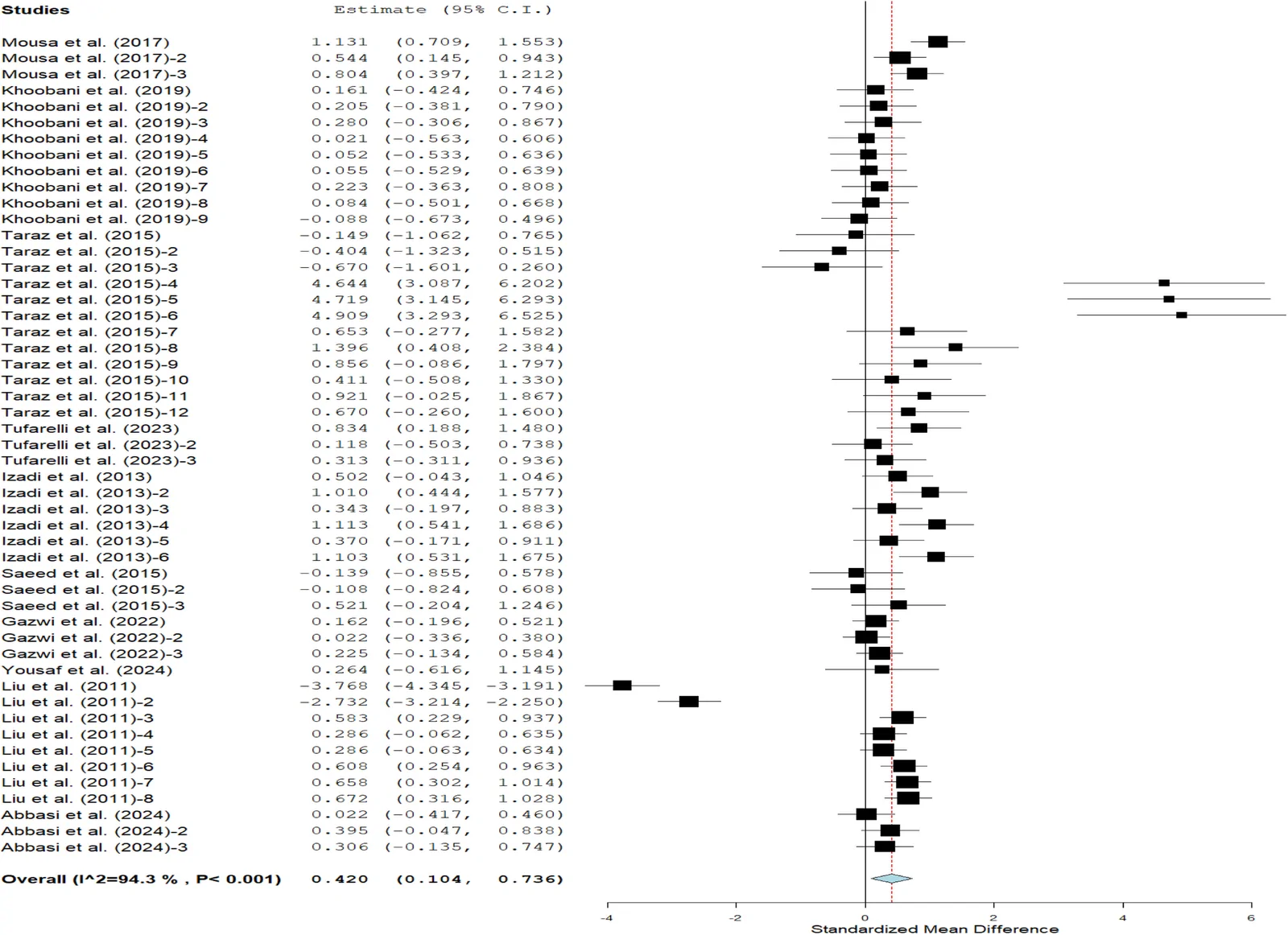
Standard mean difference in feed intake between dietary chicory and control groups.

**Fig. 3:**
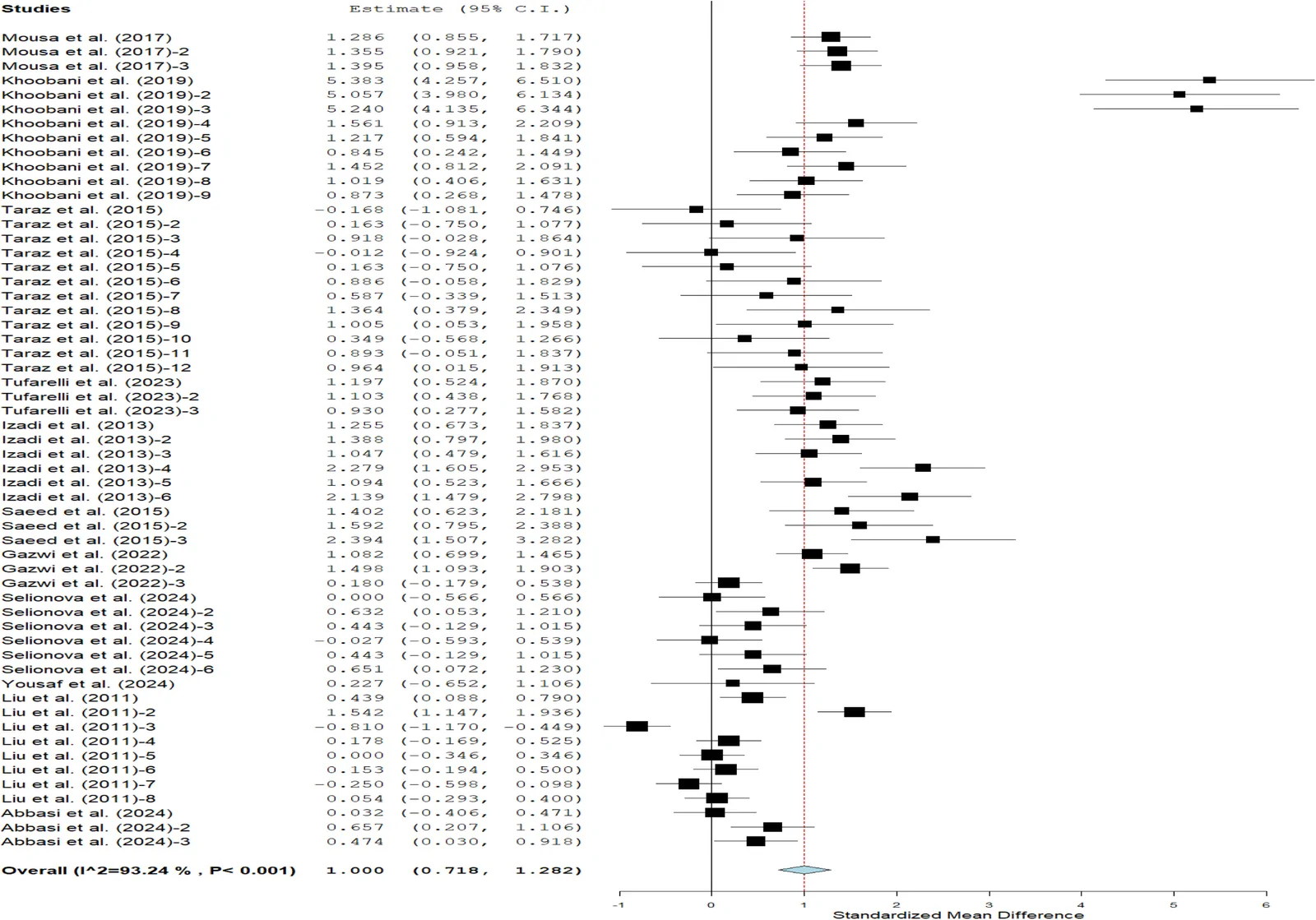
Standard mean difference in body weight gain between dietary chicory and control groups.

**Fig. 4:**
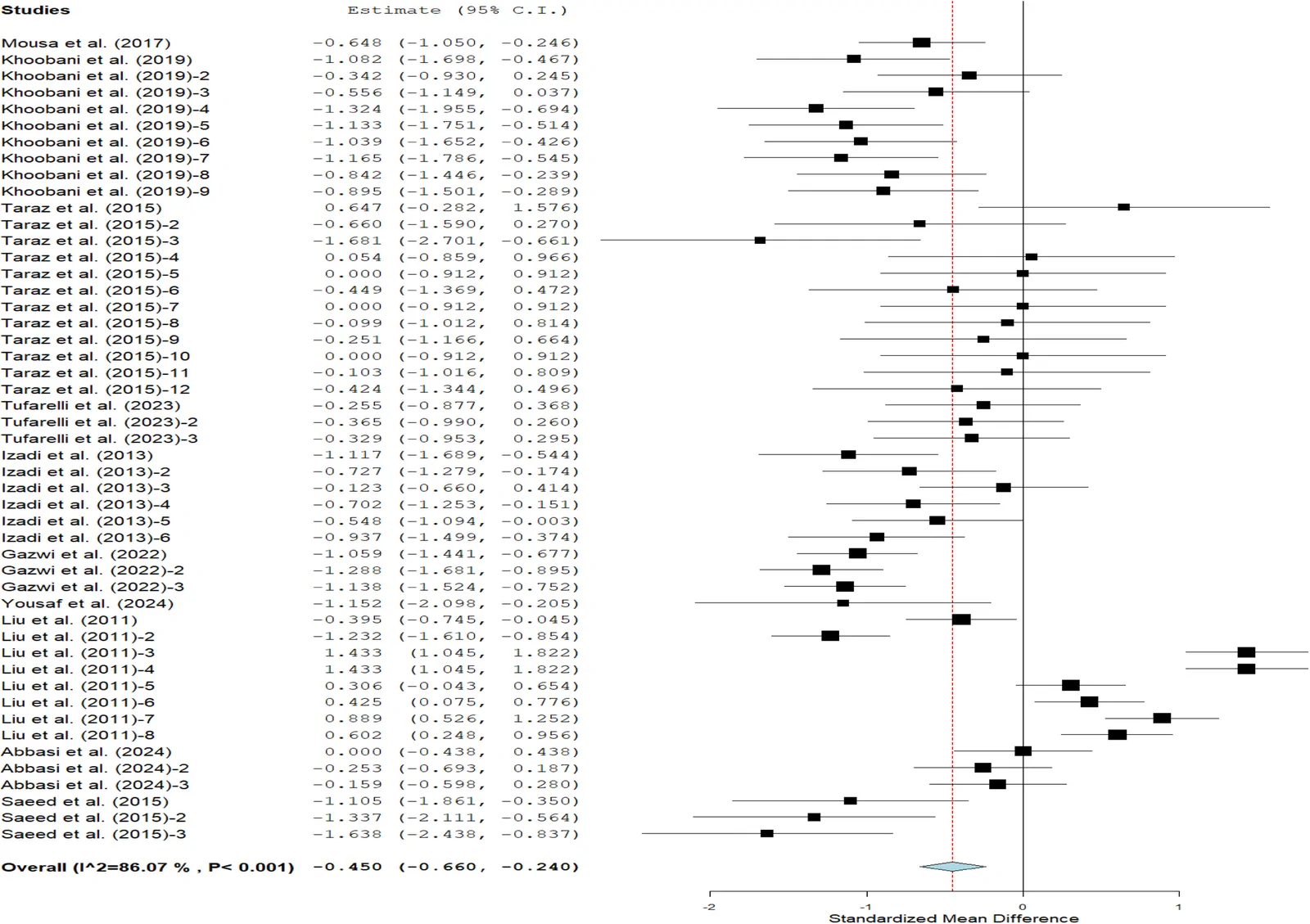
Standard mean difference in feed conversion ratio between dietary chicory and control groups.

#### Subgroup analyses

Table 2 revealed that subgroup analyses indicated varied results across examined moderators for FI in broiler chickens fed diets containing chicory. African and Asian studies produced a significant positive estimate for FI, whereas European studies showed no significant difference from the control. Broiler chickens supplemented with chicory at 0–0.9 g/kg, 1–20 g/kg, and 21–40 g/kg had improved FI, while those fed >40 g/kg had comparable FI to the control. Dietary chicory did not influence FI at the starter phase but increased FI at the grower, finisher, and overall phases. Broiler chickens fed diets containing chicory extract had comparable FI, while those offered chicory in powder form had higher FI compared to the control group. Broiler chickens supplemented with chicory via feed had increased FI compared to the control, whereas supplementing with chicory via drinking water did not influence FI. Dietary chicory did increase FI in both male and mixed flocks. Similarly, dietary chicory increased FI only in Ross.

**Table 2:** Effect of dietary chicory on the feed intake of broiler chickens.

Moderators	Subgroup	*n*	SMD	95% CI		SE	p-value
				Lower	Upper		
Continent	Africa	2	0.470	0.133	0.807	0.172	0.006
	Asia	6	0.588	0.262	0.915	0.167	< 0.001
	Europe	2	−0.190	−1.096	0.717	0.462	0.682
Inclusion level (g/kg)	0-0.9	2	1.121	0.233	2.009	0.453	0.013
	1-20	6	0.361	0.204	0.519	0.080	< 0.001
	21-40	2	0.558	0.135	0.980	0.216	0.010
	>40	1	−0.416	−1.634	0.802	0.621	0.503
Growth stage	Starter	8	−0.154	−0.802	0.494	0.331	0.642
	Grower	5	1.977	0.488	3.466	0.760	0.009
	Finisher	8	0.387	0.061	0.713	0.166	0.020
	Overall	9	0.401	0.262	0.540	0.071	< 0.001
Form	Extract	4	0.507	−0.165	1.179	0.343	0.139
	Powder	6	0.422	0.270	0.574	0.078	< 0.001
Inclusion route	Feed	8	0.450	0.104	0.795	0.176	0.011
	Water	2	0.120	−0.255	0.496	0.192	0.531
Sex	Male	4	0.603	0.009	1.197	0.303	0.047
	Mixed	6	0.283	0.130	0.435	0.078	< 0.001
Strain	Hubbard	1	0.264	−0.616	1.145	0.449	NA
	Ross	8	0.450	0.104	0.795	0.176	0.011
	Unknown	1	0.088	−0.330	0.506	0.213	0.680

*n* = number of studies; SMD = standardized mean difference; CI = confidence interval; SE = standard error; p-value = probability value.

Table 3 presents the results of the effect of the studied moderators on the BWG of broiler chickens fed diets containing chicory. Dietary chicory increased BWG in African, Asian, and European studies. Inclusion of chicory at 0–0.9 g/kg, 1–20 g/kg, and 21–40 g/kg increased BWG, while >40 g/kg had comparable BWG with the control group. Dietary chicory did increase BWG across all the growth stages. Broiler chickens fed chicory, supplemented in extract and powder forms, increased BWG. Broiler chickens supplemented with chicory via feed and drinking water had increased BWG compared to the control. Male and mixed broiler chickens offered diets containing chicory had increased BWG compared to the control group. Dietary chicory increased BWG only in Ross, Smena-9, and unknown strains.

**Table 3:** Effect of dietary chicory on the body weight gain of broiler chickens.

Moderators	Subgroup	*n*	SMD	95% CI		SE	p-value
				Lower	Upper		
Continent	Africa	2	1.124	0.723	1.525	0.205	< 0.001
	Asia	6	1.318	0.885	1.752	0.221	< 0.001
	Europe	3	0.366	0.083	0.648	0.144	0.011
Inclusion level (g/kg)	0-0.9	3	0.574	0.349	0.799	0.115	< 0.001
	1-20	6	1.719	1.153	2.284	0.289	< 0.001
	21-40	2	1.133	0.400	1.866	0.374	0.002
	>40	1	0.160	−0.298	0.619	0.234	0.493
Growth stage	Starter	9	1.337	0.501	2.173	0.427	0.002
	Grower	6	0.812	0.376	1.248	0.223	< 0.001
	Finisher	5	1.085	0.810	1.359	0.140	< 0.001
	Overall	8	0.790	0.460	1.121	0.169	< 0.001
Form	Extract	5	0.546	0.310	0.783	0.121	< 0.001
	Powder	6	1.570	1.047	2.092	0.267	< 0.001
Inclusion route	Feed	9	0.972	0.676	1.269	0.151	< 0.001
	Water	2	1.405	0.551	2.259	0.436	0.001
Sex	Male	7	0.625	0.355	0.896	0.138	< 0.001
	Mixed	4	1.395	0.901	1.888	0.252	< 0.001
Strain	Hubbard	1	0.227	−0.652	1.106	0.449	NA
	Ross	8	1.056	0.726	1.387	0.169	< 0.001
	Smena-9	1	0.352	0.111	0.594	0.123	0.004
	Unknown	1	1.762	1.197	2.327	0.288	< 0.001

*n* = number of studies; SMD = standardized mean difference; CI = confidence interval; SE = standard error; p-value = probability value.

African and Asian studies produced a significant negative estimate for FCR, whereas European studies showed no significant difference from the control (Table 4). Dietary chicory at 0–0.9 g/kg, 1–20 g/kg, and 21–40 g/kg levels reduced FCR, while >40 g/kg had comparable FCR with the control group. Dietary chicory reduced FCR only at the grower, finisher, and overall phases. Broiler chickens fed diets containing chicory extract had comparable FCR, while those offered chicory in powder form had lower FCR compared to the control group. Broiler chickens supplemented with chicory via feed and drinking water had reduced FCR compared to the control. Only the mixed flocks produced lower FCR. Dietary chicory reduced FCR only in Ross and unknown strains.

**Table 4:** Effect of dietary chicory on the feed conversion ratio of broiler chickens.

Moderators	Subgroup	*n*	SMD	95% CI		SE	p-value
				Lower	Upper		
Continent	Africa	2	−1.036	−1.299	−0.774	0.134	< 0.001
	Asia	6	−0.635	−0.807	−0.463	0.088	< 0.001
	Europe	2	0.241	−0.256	0.738	0.254	0.342
Inclusion level (g/kg)	0-0.9	2	−0.503	−0.832	−0.173	0.168	0.003
	1-20	6	−0.808	−0.988	−0.628	0.092	< 0.001
	21-40	2	−0.427	−0.723	−0.131	0.151	0.005
	>40	1	0.432	−0.192	1.056	0.318	0.175
Growth stage	Starter	8	−0.373	−0.816	0.070	0.226	0.099
	Grower	4	−0.301	−0.537	−0.066	0.120	0.012
	Finisher	4	−0.845	−1.248	−0.443	0.205	< 0.001
	Overall	9	−0.465	−0.803	−0.127	0.172	0.007
Form	Extract	4	−0.252	−0.605	0.102	0.180	0.163
	Powder	6	−0.646	−0.811	−0.482	0.084	< 0.001
Inclusion route	Feed	9	−0.382	−0.597	−0.167	0.110	< 0.001
	Water	2	−1.314	−1.718	−0.909	0.207	< 0.001
Sex	Male	4	−0.112	−0.387	0.162	0.140	0.423
	Mixed	6	−0.923	−1.087	−0.760	0.083	< 0.001
Strain	Hubbard	1	−1.152	−2.098	−0.205	0.483	NA
	Ross	8	−0.382	−0.597	−0.167	0.110	< 0.001
	Unknown	1	−1.350	−1.798	−0.902	0.229	< 0.001

*n* = number of studies; SMD = standardized mean difference; CI = confidence interval; SE = standard error; p-value = probability value.

#### Meta-regression analyses

The results of the meta-regression analyses indicated that only the inclusion level and growth stage influenced the FI of broiler chickens fed diets containing chicory (Table 5). Likewise, continent, inclusion level, form, and sex influenced the BWG of broiler chickens fed diets containing chicory. Furthermore, continent, inclusion level, form, inclusion route, and sex influenced the FCR of broiler chickens reared on dietary chicory.

**Table 5:** Moderators that influenced growth performance indices of broiler chickens fed diets with or without chicory.

Variables	Moderators	SMD	Q_M_	df	p-value	*I^2^* (%)	R^2^ (%)
Feed intake (g)	Continent	−0.185	4.338	2	0.114	93.92	4.07
	Inclusion level (g/kg)	0.330	8.876	3	0.031	93.59	8.92
	Growth stage	0.383	15.237	3	0.002	93.12	16.14
	Form	0.408	0.007	1	0.935	94.41	0.00
	Inclusion route	0.786	0.533	1	0.465	97.46	0.00
	Sex	0.261	0.771	1	0.380	94.37	0.00
	Strain	0.447	0.272	2	0.873	94.68	0.00
Body weight gain (g)	Continent	0.383	9.843	2	0.007	91.94	15.64
	Inclusion level (g/kg)	1.669	23.59	3	< 0.001	90.15	32.04
	Growth stage	0.818	1.988	3	0.575	93.37	0.00
	Form	1.531	13.88	1	< 0.001	91.43	22.13
	Inclusion route	0.971	0.560	1	0.454	93.35	0.00
	Sex	1.366	7.124	1	0.008	92.41	10.93
	Strain	1.051	4.364	3	0.225	93.20	2.80
Feed conversion ratio	Continent	0.253	23.21	2	< 0.001	78.85	37.98
	Inclusion level (g/kg)	−0.829	27.93	3	< 0.001	78.16	40.63
	Growth stage	−0.465	1.937	3	0.586	86.06	0.00
	Form	−0.670	4.303	1	0.038	84.94	8.01
	Inclusion route	−0.382	5.407	1	0.020	85.09	8.64
	Sex	−0.926	19.74	1	< 0.001	80.42	33.06
	Strain	−0.382	5.384	2	0.068	85.50	6.95

SMD = standardized mean difference; Q_M_ = coefficient of moderators; df = degree of freedom; *I²* = inconsistency index; R² = proportion of heterogeneity explained by covariates.

#### Publication bias

Visual inspection of the funnel plots revealed some asymmetry across the FI, BWG, and FCR. For FI, most effect sizes clustered symmetrically around the pooled estimate at low standard errors (Fig. 5). However, a small number of studies with larger standard errors were scattered toward the extreme positive end of the plot. A similar pattern was observed for BWG, with most studies within the expected pseudo-confidence funnel (Fig. 6). Nonetheless, a few estimates extended toward the positive tail. The FCR funnel plot (Fig. 7) showed the most pronounced asymmetry. Most studies concentrated near the null effect on the left arm of the funnel, and a handful of studies with larger standard errors deviated toward the positive right tail. Despite this visual asymmetry, *N*_fs_ indicated that the pooled estimates for FI and BWG were highly robust to publication bias. Table 6 indicated that 749 and 5,535 hypothetical null studies, respectively, would be required to overturn the significance of these effects, far exceeding the conventional robustness thresholds of 60 and 65 studies, respectively. For FCR, 770 null studies would be required against a threshold of 60.

**Fig. 5:**
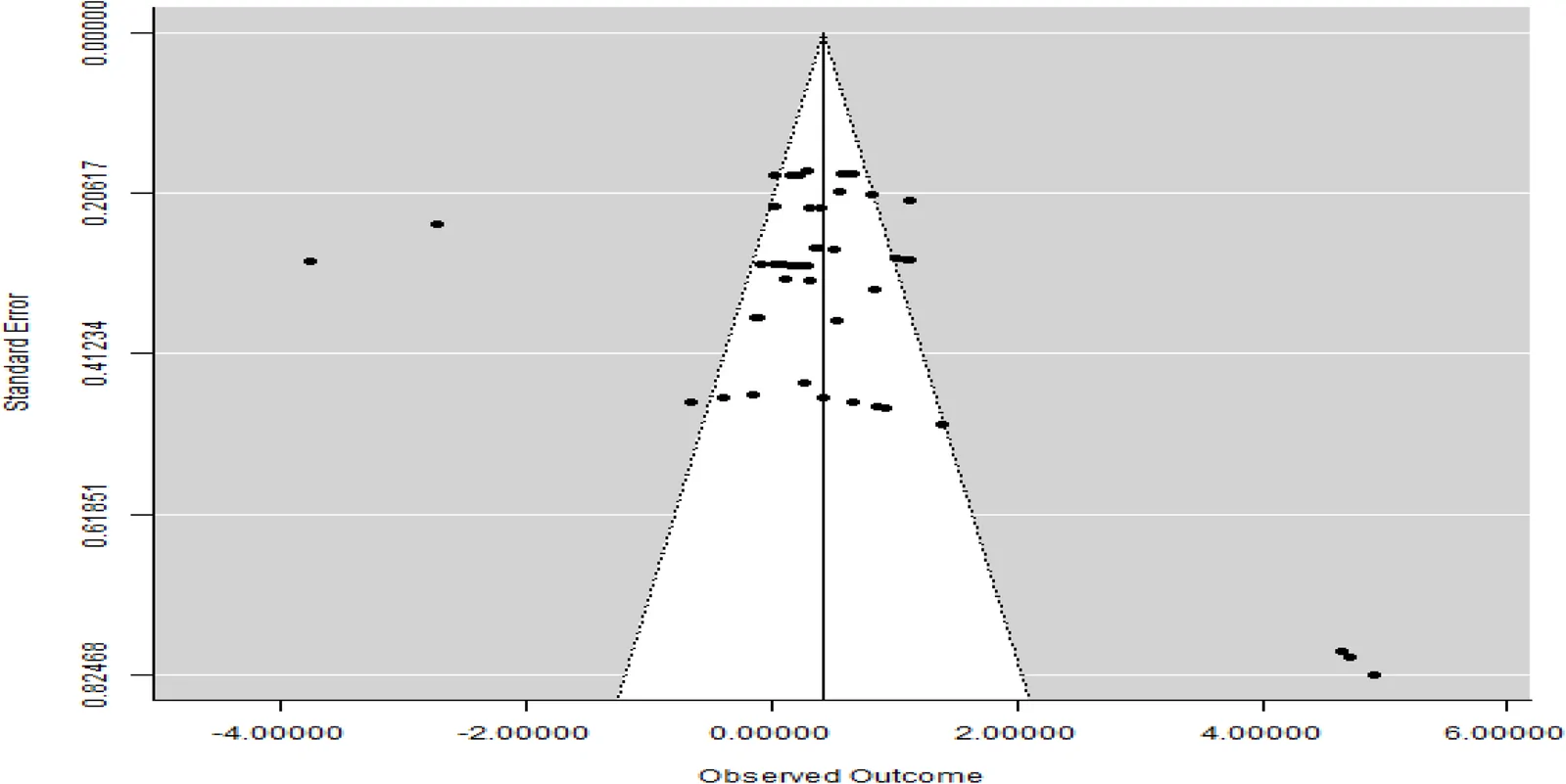
Funnel plot of feed intake of broiler chickens on diets containing chicory.

**Fig. 6:**
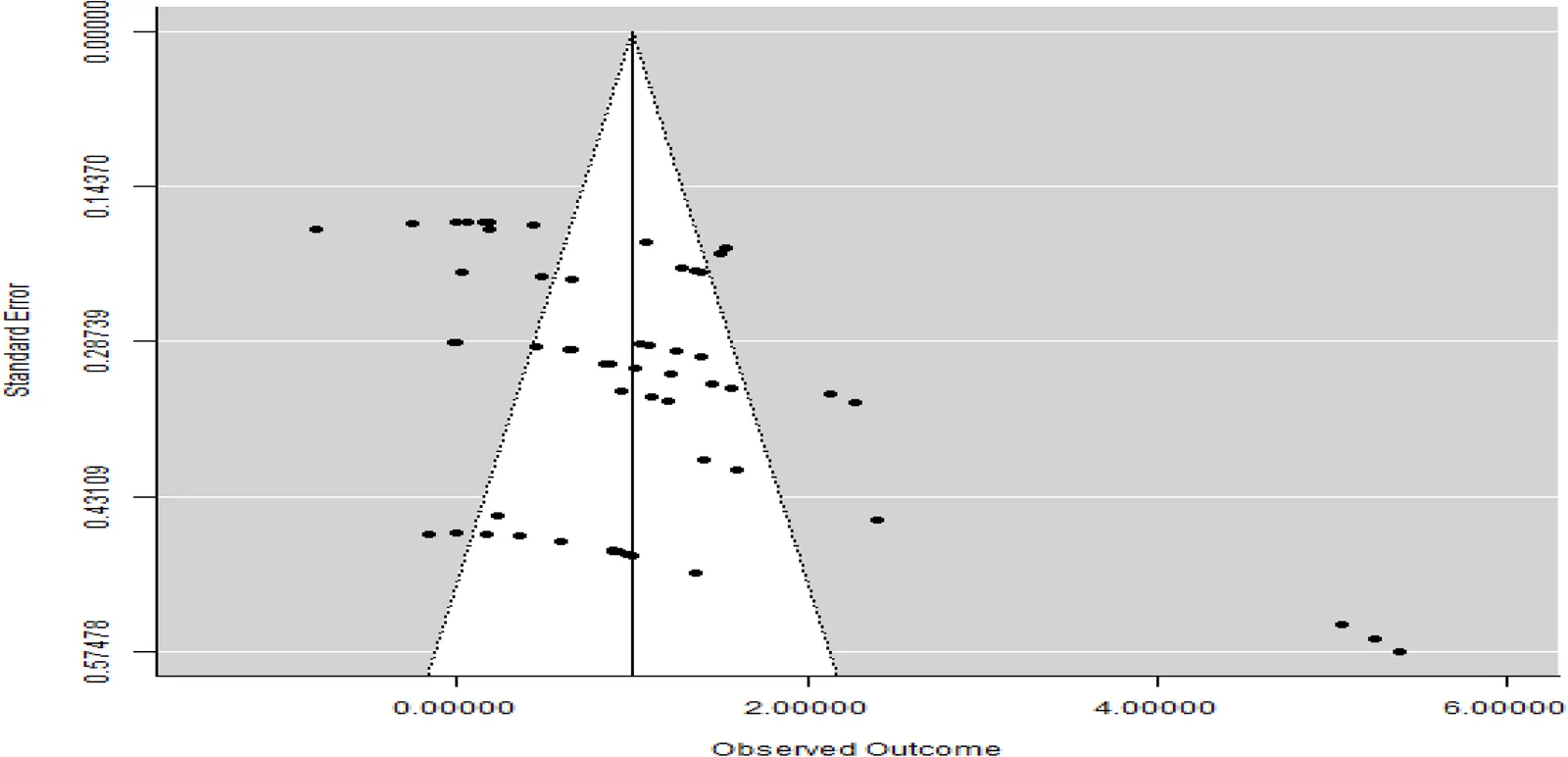
Funnel plot of body weight gain of broiler chickens on diets containing chicory.

**Fig. 7:**
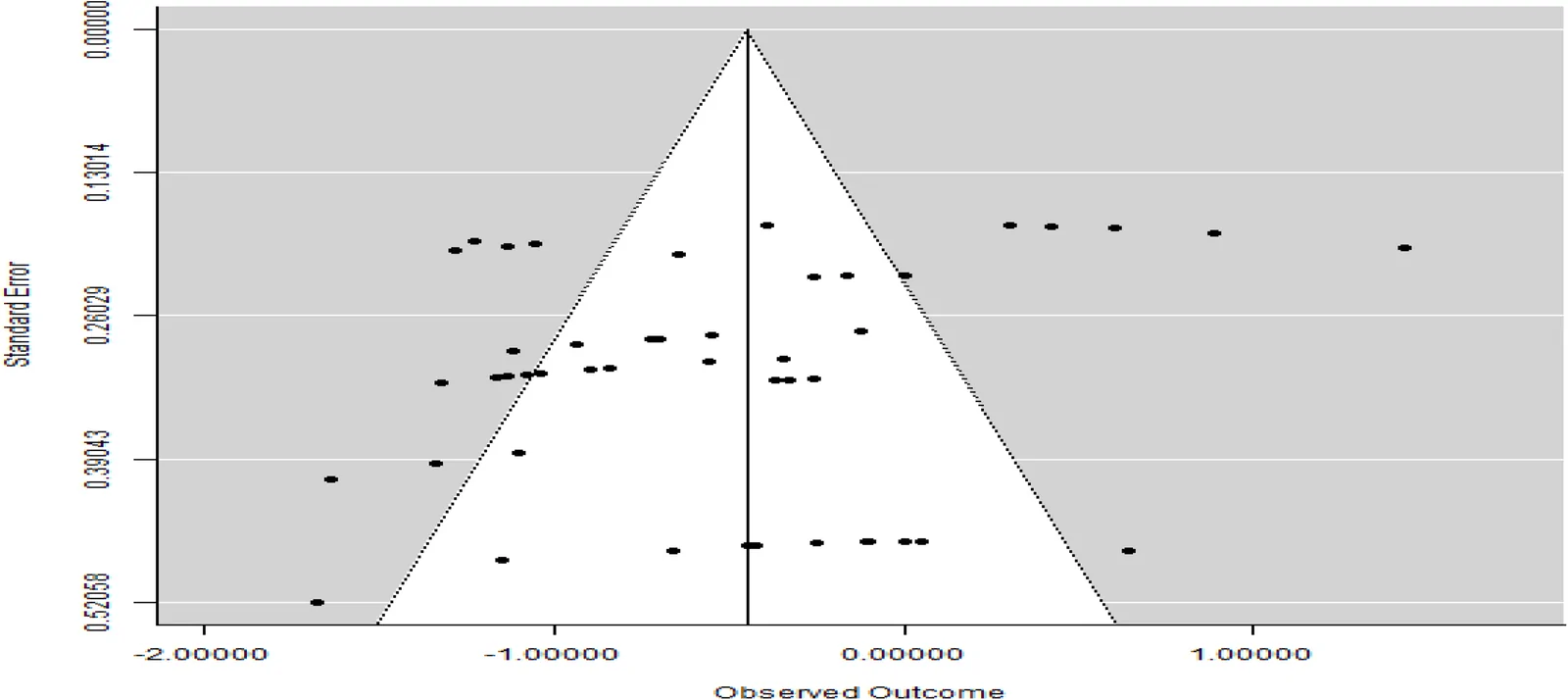
Funnel plot of feed conversion ratio of broiler chickens on diets containing chicory.

**Table 6:** Analysis of publication bias.

Outcomes	SMD	Observed significant	Target significant	Nfs number	Number of studies	Nfs>(5N_study_+10)
Feed intake (g)	0.292	< 0.001	0.05	749	10	60
Body weight gain (g)	0.710	< 0.001	0.05	5535	11	65
Feed conversion ratio	−0.310	< 0.001	0.05	770	10	60

SMD = standardized mean difference; *Nfs* = fail-safe number.

## Discussion

Poor reporting of randomisation, allocation concealment and blinding is typical of animal nutrition trials and can inflate effect sizes (Egbu and Smit, 2026). The predominance of “unclear” ratings reflects incomplete reporting rather than demonstrated bias. Feed intake, BWG, and FCR are objective measures, so detection bias is probably small. The very high heterogeneity (*I*²) may partly reflect these methodological differences, in addition to the moderators tested. Only two studies had high-risk domains, and the other nine were largely unclear rather than flawed. Hence, the pooled benefits of chicory should be interpreted cautiously until trials report randomisation, concealment and blinding explicitly, in line with the ARRIVE guidelines. Sensitivity analysis excluding studies at high risk would test whether the conclusions are robust.

The pooled analysis indicates that dietary chicory increases FI and BWG while lowering FCR in broiler chickens. This result is consistent with the mode of action of chicory bioactive constituents. Chicory is rich in inulin, which is resistant to endogenous digestive enzymes but is readily fermented by cecal and colonic microbiota into short-chain fatty acids (SCFAs) (Puhlmann et al., 2022). These SCFAs lower luminal pH, selectively favor the proliferation of beneficial taxa over pathogenic competitors, and serve as an energy substrate for colonocytes, thereby supporting mucosal integrity and nutrient absorptive capacity (Wu et al., 2019; Ali et al., 2022). This microbiota-mediated route offers a plausible mechanism for the observed increase in BWG, as improved gut integrity and a more favorable microbial ecology would be expected to enhance the efficiency with which ingested nutrients are converted into tissue accretion. The increase in FI was not anticipated, since chicory also contains bitter sesquiterpene lactones such as lactucin and lactucopicrin that might be expected to depress palatability (Guo et al., 2026). The increase in FI observed in this study likely reflects a net balance in which the benefits of inulin fermentation, such as enhanced appetite, improved digestion, and better gut health, could have outweighed any bitterness-related deterrents to palatability at the inclusion levels captured across the pooled studies. Nonetheless, the overall pooled effects occurred alongside *I*² values of 94.29%, 93.24%, and 86.07% for FI, BWG, and FCR, respectively. This high variance reflects the diverse experimental conditions across the contributing primary studies.

Continent moderated BWG and FCR but not FI, with African and Asian studies producing better estimates, whereas European studies showed a positive response for BWG only. A plausible explanation lies in regional differences in basal diet composition. Broiler diets in Europe are frequently based on wheat or barley, cereals with an inherently higher content of soluble non-starch polysaccharides. Hence, the baseline digesta viscosity is higher than that of the corn meal diets that are common in Africa and Asia (Jha and Mishra, 2021). Where baseline viscosity is already elevated, the additional fermentable fiber from chicory may contribute less marginal benefit or may even compound antinutritional effects compared to diets based on low-viscosity cereals where the fiber substrate limits baseline fermentation. Furthermore, regional variation in flock health status and antimicrobial stewardship policy (Beber et al., 2025) may modulate the baseline gut microbiota against which a prebiotic ingredient acts, with a less favorable starting microbiota offering greater scope for improvement. That continent explained comparatively little FI heterogeneity while explaining more than a third of FCR heterogeneity, suggesting that regional factors influence nutrient conversion efficiency rather than appetite drive.

Inclusion level was an influential moderator of FCR and BWG but explained comparatively little FI heterogeneity, indicating that it modulates the efficiency with which consumed feed is converted into tissue far more than the volume of feed consumed. Across all three outcomes, benefits were concentrated at 1-20 g/kg and did not extend to >40 g/kg. This pattern suggests a dose-response effect, in which bioactive compounds administered beyond an optimum can depress rather than enhance performance (Windisch et al., 2008). Mechanistically, this result is consistent with the antinutritive behavior of chicory's soluble fiber fraction. At higher inclusion, soluble non-starch polysaccharides increase digesta viscosity, slow nutrient-enzyme contact, and dilute the nutrient density of the diet, thereby offsetting the benefits observed at lower levels (Jha and Mishra, 2021). The inclusion level explained substantially more variance in BWG and FCR than in FI. This further supports that appetite is influenced by palatability and gut motility constraints. Conversely, the metabolic benefits of SCFAs toward nutrient absorption scale with fermentable substrate availability only up to the threshold where physical viscosity costs begin to dominate.

Growth stage moderated FI but not BWG or FCR, despite clear differences between subgroup means for all three outcomes. This apparent inconsistency indicates that the growth stage exhibited categorical differences that did not follow a linear trend detectable by meta-regression. Our results show that dietary chicory did not influence the FI and FCR during the starter phase. This response was likely driven by the relative immaturity of the gut microbiota and digestive enzyme complement at this stage, which limits young chicks’ capacity to ferment inulin efficiently (Ravindran and Abdollahi, 2021). While Izadi et al. (2013) did note starter phase improvements in FI and FCR, our results imply that early physiological constraints and potential palatability sensitivities to bitter sesquiterpene lactones neutralize overall starter performance. As the gut microbiota matures and cecal fermentative capacity expands with age (Rinttilä and Apajalahti, 2013), the prebiotic benefits of chicory are established and remain consistent through to market age.

Form moderated BWG and FCR but not FI, even though the FI subgroup analysis indicated that chicory powder produced a significant effect while the extract had no significant effect. This discrepancy likely reflects the wide CI and comparatively small extract subgroup for FI, which reduces the statistical power of the categorical moderator test even where a difference in estimates may exist. Where form did reach significance, powder consistently outperformed extract. Chicory powder retains the structural fiber and sesquiterpene lactone fractions (Puhlmann et al., 2024), whereas extraction concentrates bioactive compounds while isolating them from the structural fiber that slows down degradation (Jha and Mishra, 2021). A more gradual and matrix-buffered release of fermentable substrate from powder, relative to the concentrated and immediately bioavailable but depleted fiber extract, offers a plausible explanation for its stronger and more consistent effect on both BWG and FCR.

The inclusion route did not moderate FI or BWG, although the BWG subgroup showed both routes to be independently effective. This response indicates that the two routes achieve a comparable growth-promoting effect through the same inherent gut-microbiota mechanism. Route did moderate FCR, and the FCR subgroup estimates confirm this, with the water estimates roughly three and a half times larger in magnitude. A plausible mechanism is that continuous and low-concentration exposure to chicory bioactive compounds via drinking water sustains an even substrate supply. Because broilers exhibit a frequent and intermittent drinking pattern throughout the day, water delivery avoids the pronounced physiological bolus effect associated with feed (Dorado-Montenegro et al., 2024). This steady microdosing provides a constant supply of fermentable substrates to the hindgut, potentially stabilizing SCFAs production and maintaining low luminal pH (Sadati et al., 2022). This interpretation warrants caution, however, because the water-route studies in this dataset are drawn exclusively from Asia. Consequently, route and continent are confounded in the present evidence base, meaning the apparent superiority of water delivery cannot be fully isolated from regional environmental or management effects with only two water-route studies available.

Furthermore, sex did not moderate FI but moderated BWG and FCR, with mixed-sex flocks showing a larger pooled improvement than male flocks for both outcomes. Male and female broilers differ inherently in growth trajectory, metabolic rate, and carcass composition. Males typically exhibit greater feed intake and lean tissue accretion driven partly by higher circulating androgens, whereas females tend to exhibit lower baseline nutrient digestibility and a higher predisposition for fat deposition (Ebrahimi et al., 2026). Because chicory's efficiency benefit appears to operate through improved gut integrity and nutrient absorption, it plausibly delivers a relative improvement in mixed-sex compositions where female birds provide a baseline scope for physiological optimization (Ebrahimi et al., 2026). Additionally, distinct social feeding behaviors and competitive dynamics inherent to commingled rearing may further modulate this response in mixed-sex flocks (England et al., 2022). This finding represents a case in which the moderator likely reflects an underlying biological sex-by-treatment interaction rather than simply flagging an arbitrary bird-management difference.

Strain was a significant moderator of FI in the subgroup analysis, yet strain did not reach significance in the FI meta-regression, nor did it in the BWG or FCR models. Across all three outcomes, the strain categories with the smallest sample sizes carry the widest CI. This limitation restricts the ability of the categorical moderator test to detect a genuine strain effect even where the estimates differ. The observed consistent response documented in Ross is nonetheless consistent with this strain's decades-long selection history for high feed intake capacity and rapid, efficient lean tissue accretion (Tavarez & Solis de los Santos, 2016). Because these modern genetic lines possess accelerated metabolic demands, they are sensitive to subclinical intestinal stress. Consequently, they display a receptive physiological framework for functional carbohydrates and fiber structures, such as inulin, which stabilize gut health, lower luminal pH, and maximize nutrient extraction to meet the strain's elevated genetic growth potential (M’Sadeq et al., 2015).

With respect to the robustness of the evidence base, the *N*_fs_ obtained for all three outcomes indicate that an implausibly large number of unpublished null-result studies would be required to reverse these pooled conclusions. This provides reassurance that the observed benefits of chicory are unlikely to be artifacts of selective publication (Rosenberg, 2005). Nonetheless, the mild asymmetry visible in all three funnel plots warrants some caution. Funnel plot asymmetry can arise from genuine heterogeneity in experimental conditions as readily as from publication bias. Furthermore, formal asymmetry tests are well documented to have limited statistical power when, as here, fewer than fifteen studies are pooled per outcome (Sterne et al., 2011).

Nevertheless, the evidence base was restricted to 11 studies, and several subgroup categories were each represented by a single study, producing highly imprecise estimates within these strata. Even after accounting for the significant moderators, residual heterogeneity remained very high across all three outcomes. This implies that unmeasured factors, such as precise phytochemical concentration of the chicory preparations, basal diet formulation, and environmental or management conditions, likely contributed additional variability that the tested moderators could not capture. The confounding between the inclusion route and continent in the FCR dataset suggests that route-specific recommendations should be treated as provisional until they are tested in a more geographically balanced set of primary trials. The exclusive inclusion of unchallenged trials, while methodologically appropriate for isolating chicory's baseline growth-promoting effect, limits extrapolation to the disease- or stress-challenged conditions typical of commercial farming. Finally, the biological mechanisms proposed here are inferred from the chicory and inulin literature rather than measured directly within the primary trials pooled in this analysis. Hence, they should be treated as plausible physiological explanations rather than confirmed mechanisms for this specific dataset.

## Conclusion

This study represents the first quantitative synthesis to demonstrate that dietary chicory concurrently improves feed intake, body weight gain, and feed efficiency in broiler chickens. This study provides poultry nutritionists and feed formulators with an evidence-based framework for incorporating chicory into antibiotic-free broiler diets. Overall results indicate that a powder form supplemented at 1-20 g/kg from the grower phase onwards is effective. Future work should explore additional factors to explain variability and more primary trials across understudied strains and regions to close the remaining gaps in this evidence base.

## CRediT authorship contribution statement

Christiaan J. Smit: conceived and designed the study; acquired, analyzed, and interpreted the data; drafted the manuscript and reviewed it critically for crucial intellectual content; gave final approval of the version to be published; and agrees to be accountable for all aspects of the work in ensuring that questions related to the accuracy or integrity of any part of the work are appropriately investigated and resolved.

## Disclosures

The authors declare that they have no known competing financial interests or personal relationships that could have appeared to influence the work reported in this paper.

## Footnotes

Supplementary material associated with this article can be found, in the online version, at doi:10.1016/j.psj.2026.107956.

## References

[bib0001] Abbasi B.H.A., Rashid N., Bilal R.M., Wadaan M.A., Khan M.F. (2024). Supplementation of *cichorium intybus* roots improved the growth performance, immunity response, gut ecology and morphology of broilers chicken Ross 308 strain. J. King Saud Univ. Sci..

[bib0002] Abo Ghanima M.M., Aljahdali N., Abuljadayel D.A., Shafi M.E., Qadhi A., Abd El-Hack M.E., Mohamed L.A. (2023). Effects of dietary supplementation of amla, chicory, and leek extracts on growth performance, immunity and blood biochemical parameters of broilers. Ital. J. Anim. Sci..

[bib0003] Akram H., Zafar H., Abbasi B.H. (2025). The chicory root (*Cichorium intybus* var. sativum) frontier: pioneering biotechnological advancements. Phytochem. Rev..

[bib0004] Ali Q., Ma S., La S., Guo Z., Liu B., Gao Z., Farooq U., Wang Z., Zhu X., Cui Y., Li D., Shi Y. (2022). Microbial short-chain fatty acids: a bridge between dietary fibers and poultry gut health—a review. Anim. Biosci..

[bib0005] Beber C.L., Aragrande M., Canali M. (2025). Policies and strategies to control antimicrobial resistance in livestock production: a comparative analysis of national action plans in European Union Member States. Health Policy.

[bib0006] Bhandari M., Lokesh D., Thenissery A., Shrestha R., Rajashekara G. (2026). Antimicrobial peptides isolated from probiotics as an alternative to antibiotics against *Salmonella* infection. Appl. Environ. Microbiol..

[bib0007] Boltz T.P., Ward N.E., Ayres V.E., Lamp A.E., Moritz J.S. (2020). The effect of varying steam conditioning temperature and time on pellet manufacture variables, true amino acid digestibility, and feed enzyme recovery. J. Appl. Poult. Res..

[bib0008] Bungau S., Tit D.M., Behl T., Aleya L., Zaha D.C. (2021). Aspects of excessive antibiotic consumption and environmental influences correlated with the occurrence of resistance to antimicrobial agents. Curr. Opin. Environ. Sci. Health.

[bib0009] Cao S., Yang W., Zhu X., Liu C., Lu J., Si Z., Pei L., Zhang L., Hu W., Li Y., Wang Z., Pang Z., Xue X., Li Y. (2022). Isolation and identification of the broad-spectrum high-efficiency phage vB_SalP_LDW16 and its therapeutic application in chickens. BMC Vet. Res..

[bib0010] Chukwudi P., Umeugokwe P.I., Ikeh N.E., Amaefule B.C. (2025). The effects of organic acids on broiler chicken nutrition: a review. Anim. Res. One Health.

[bib0011] Dorado-Montenegro S., Habibi M.F., Gerrits W.J., de Vries S. (2024). Effect of adding soluble viscous fibers to diets containing coarse and finely ground insoluble fibers on digesta transit behavior and nutrient digestibility in broiler chickens. Poult. Sci..

[bib0012] Ebrahimi M., Daneshyar M., Payvastegan S., Ahmadi H. (2026). Association and variation in performance, nutrient digestibility, carcass traits, and intestinal development of broiler chickens by various male-to-female ratios. Vet. Anim. Sci..

[bib0013] Egbu C.F., Smit C.J. (2026). Dietary licorice improves carcass yield, hepatocellular protection, gut health, oxidative status, and cecal bacterial balance in broiler chickens. Vet. Anim. Sci..

[bib0014] England A.D., Gharib-Naseri K., Kheravii S.K., Wu S.B. (2022). Rearing broilers as mixed or single-sex: relevance to performance, coefficient of variation, and flock uniformity. Poult. Sci..

[bib0015] Garritty C., Hamel C., Trivella M., Gartlehner G., Nussbaumer-Streit B., Devane D., Kamel C., Griebler U., King V.J. (2024). Updated recommendations for the Cochrane rapid review methods guidance for rapid reviews of effectiveness. BMJ.

[bib0016] Gazwi H.S.S., Mahmoud M.E., Toson E.M.A. (2022). Analysis of the phytochemicals of *coriandrum sativum* and *cichorium intybus* aqueous extracts and their biological effects on broiler chickens. Sci. Rep..

[bib0017] Guo X., Cheng Y., Qiao Z., Hu Z., Xu J., Song W., Qi J., Zeng X., Yuan Y., Ling L., Yue T. (2026). Chicory-derived lactucins: research progress on its biological activity and extraction methods. Food Chem..

[bib0018] Hooijmans C.R., Rovers M.M., De Vries R.B., Leenaars M., Ritskes-Hoitinga M., Langendam M.W. (2014). SYRCLE’s risk of bias tool for animal studies. BMC Med. Res. Methodol..

[bib0019] Izadi H., Arshami J., Golian A., Raji M.R. (2013). Effects of chicory root powder on growth performance and histomorphometry of jejunum in broiler chicks. Vet. Res. Forum.

[bib0020] Jha R., Mishra P. (2021). Dietary fiber in poultry nutrition and their effects on nutrient utilization, performance, gut health, and on the environment: a review. J. Anim. Sci. Biotechnol..

[bib0021] Khoobani M., Hasheminezhad S.H., Javandel F., Nosrati M., Seidavi A., Kadim I.T., Laudadio V., Tufarelli V. (2019). Effects of dietary chicory (*Chicorium intybus* L.) and probiotic blend as natural feed additives on performance traits, blood biochemistry, and gut microbiota of broiler chickens. Antibiotics.

[bib0022] Khosravi A., Boldaji F., Dastar B., Torshizi M.A.K., Alemi M., Hoseinifar S.H. (2025). A synbiotic improves performance and gut health in broiler chickens. Sci. Rep..

[bib0023] Korver D.R. (2023). Current challenges in poultry nutrition, health, and welfare. Animal.

[bib0024] Liu H.Y., Ivarsson E., Jönsson J., Holm L., Lundh T., Lindberg J.E. (2011). Growth performance, digestibility, and gut development of broiler chickens on diets with inclusion of chicory (*Cichorium intybus* L.). Poult. Sci..

[bib0026] M’Sadeq S.A., Wu S.B., Swick R.A., Choct M. (2015). Dietary acylated starch improves performance and gut health in necrotic enteritis challenged broilers. Poult. Sci..

[bib0025] Mousa M.A., Osman A.S., Hady H.A. (2017). Performance, immunology and biochemical parameters of *Moringa oleifera* and/or *cichorium intybus* addition to broiler chicken ration. J. Vet. Med. Anim. Health.

[bib0027] Pesti G.M., Billard L., Wu S.B., Morgan N.K., Taylor P.S., de Las Heras-Saldana S. (2023). Partition of variation for predicting experimental power with a broiler chicken example. Poult. Sci..

[bib0028] Puhlmann M.L., Jokela R., van Dongen K.C.W., Bui T.P.N., van Hangelbroek R.W.J., Smidt H., de Vos W.M., Feskens E.J.M. (2022). Dried chicory root improves bowel function, benefits intestinal microbial trophic chains and increases faecal and circulating short chain fatty acids in subjects at risk for type 2 diabetes. Gut Microbiome.

[bib0029] Puhlmann M.L., van de Rakt E., Kerezoudi E.N., Rangel I., Brummer R.J., Smidt H., Kaper F.S., de Vos W.M. (2024). Analysis of the fermentation kinetics and gut microbiota modulatory effect of dried chicory root reveals the impact of the plant-cell matrix rationalizing its conversion in the distal colon. Microbiome Res. Rep..

[bib0030] Rafeeq M., Bilal R.M., Alagawany M., Batool F., Yameen K., Farag M.R., Ali S., Elsner S.S., El-Shall N.A. (2022). The use of some herbal plants as effective alternatives to antibiotic growth enhancers in poultry nutrition. World’s Poult. Sci. J..

[bib0031] Ramlucken U., Ramchuran S.O., Moonsamy G., van Rensburg C.J., Thantsha M.S., Lalloo R. (2021). Production and stability of a multi-strain *Bacillus* based probiotic product for commercial use in poultry. Biotechnol. Rep..

[bib0032] Ravindran V., Abdollahi M.R. (2021). Nutrition and digestive physiology of the broiler chick: state of the art and outlook. Animals.

[bib0033] Rehman A., Arif M., Sajjad N., Al-Ghadi M.Q., Alagawany M., Abd El-Hack M.E., Alhimaidi A.R., Elsner S.S., Almutairi B.O., Amran R.A., Hussein E.O.S., Swelum A.A. (2020). Dietary effect of probiotics and prebiotics on broiler performance, carcass, and immunity. Poult. Sci..

[bib0034] Rinttilä T., Apajalahti J. (2013). Intestinal microbiota and metabolites—implications for broiler chicken health and performance. J. Appl. Poult. Res..

[bib0035] Rosenberg M.S. (2005). The file-drawer problem revisited: a general weighted method for calculating fail-safe numbers in meta-analysis. Evolution.

[bib0036] Sadati M.M., Rezaeipour V., Abdullahpour R. (2022). Efficacy of whole wheat grain in combination with acidified drinking water on growth performance, gizzard development, intestinal morphology, and microbial population of broiler chickens. Livest. Sci..

[bib0037] Saeed M., Baloch A.R., Wang M., Soomro R.N., Baloch A.M., Bux B.A., Arian M.A., Faraz S., Zakriya H.M. (2015). Use of *Cichorium intybus* leaf extract as growth promoter, hepatoprotectant and immune modulent in broilers. J. Anim. Prod. Adv..

[bib0038] Selionova M.I., Trukhachev V.I., Zagarin A.Y., Kulikov E.I., Dmitrenko D.M., Martynova V.N., Kravchenko A.K., Vertiprakhov V.G. (2024). Expression of genes related to meat productivity, metabolic and morphological significance of broiler chickens with the use of nutritional phytochemicals. Animals.

[bib0039] Sterne J.A.C., Sutton A.J., Ioannidis J.P.A., Terrin N., Jones D.R., Lau J., Carpenter J., Rücker G., Harbord R.M., Schmid C.H., Tetzlaff J., Deeks J.J., Peters J., Macaskill P., Schwarzer G., Duval S., Altman D.G., Moher D., Higgins J.P.T. (2011). Recommendations for examining and interpreting funnel plot asymmetry in meta-analyses of randomised controlled trials. BMJ.

[bib0040] Taraz Z., Shams Shargh M., Samadi F., Ebrahimi P., Zerehdaran S. (2015). Effect of chicory plant (*Cichorium intybus* L.) extract on performance and blood parameters in broilers exposed to heat stress with emphasis on antibacterial properties. Poult. Sci. J..

[bib0041] Tavárez M.A., Solis de los Santos F. (2016). Impact of genetics and breeding on broiler production performance: a look into the past, present, and future of the industry. Anim. Front..

[bib0042] Tufarelli V., Ghavami N., Nosrati M., Rasouli B., Kadim I.T., Suarez Ramirez L., Gorlov I., Slozhenkina M., Mosolov A., Seidavi A., Ayasan T., Laudadio V. (2023). The effects of peppermint (*Mentha piperita* L.) and chicory (*Cichorium intybus* L.) in comparison with a prebiotic on productive performance, blood constituents, immunity and intestinal microflora in broiler chickens. Anim. Biotechnol..

[bib0043] Wanasawaeng W., Thomrongsuwannakij T., Chansiripornchai N. (2025). Isolation, characterization, and application of bacteriophages for *Salmonella* control in broiler chickens. Vet. Med. Int..

[bib0044] Windisch W., Schedle K., Plitzner C., Kroismayr A. (2008). Use of phytogenic products as feed additives for swine and poultry. J. Anim. Sci..

[bib0045] Wu X.Z., Wen Z.G., Hua J.L. (2019). Effects of dietary inclusion of *Lactobacillus* and inulin on growth performance, gut microbiota, nutrient utilization, and immune parameters in broilers. Poult. Sci..

[bib0046] Yousaf M., Sarwar M., Ahmed I., Kamran Z., Fouad D., Ashraf S., Ataya F.S., Ahmad S., Rahman A., Mahmood M., Khan A.K., Ahmad H.I. (2025). Effect of aqueous extract of *cichorium intybus* as alternative antibiotic growth promotor on growth performance, lipid and mineral profiles of broilers. Pak. J. Zool..

